# Three-Dimensional Separate and Joint Inversions of Multi-Component Frequency-Domain Airborne Electromagnetic Data: Synthetic Model Studies

**DOI:** 10.3390/s23156842

**Published:** 2023-08-01

**Authors:** Jun Yang, Xin Huang, Liangjun Yan, Xiaoyue Cao

**Affiliations:** Key Laboratory of Exploration Technologies for Oil and Gas Resources (Yangtze University), Ministry of Education, Wuhan 430100, China; yangj990320@163.com (J.Y.); yljemlab@163.com (L.Y.); caoxy_em@163.com (X.C.)

**Keywords:** AEM, finite element method, 3D joint inversion, multicomponent

## Abstract

Airborne electromagnetic (AEM) surveys using airborne mobile platforms enable rapid and efficient exploration of areas where groundwork is difficult. They have been widely used in fields such as shallow resource exploration and environmental engineering. Three-dimensional AEM inversion is the main technique used in fine structural interpretation. However, most current methods focus on separate component data inversions, which limit the kinds of structures that can be recovered in the inversion results. To address this issue, a method for the robust 3D joint inversion of multicomponent frequency-domain AEM data was developed in this study. First, a finite element method based on unstructured tetrahedral grids was used to solve the forward problem of frequency-domain AEM data for both isotropic and anisotropic media. During inversion, a limited-memory quasi-Newton (L-BFGS) method was used to reduce the memory requirements and enable the joint inversion of large-scale multicomponent AEM data. The effectiveness of our algorithm was demonstrated using synthetic models for both isotropic and anisotropic cases, with 5% Gaussian noise added to the modeling data to simulate the measured data for separate and joint inversions. The results of the synthetic models show that joint inversion has advantages over separate inversion in that it enables the recovery of finer underground structures and provides a novel approach for the fine interpretation of frequency-domain AEM data.

## 1. Introduction

The rapid development of the economy has led to a continuous increase in demand for minerals, with an urgent need to expand resource exploration to areas with complex geological conditions and conduct secondary fine detection in near-surface areas. The AEM method, which uses airborne mobile platforms, has advantages in terms of fast exploration speed, low cost, and strong adaptability to terrain and landforms. It can quickly complete regional electromagnetic data scanning and collection work, and has become one of the preferred technical methods for exploration work in areas with complex geological conditions [1,2,3]. In addition, with the continuous upgrading of AEM hardware systems, their lateral resolution and sampling rates have been improved considerably, providing the necessary guarantees for fine near-surface detection. However, the increasing complexity and refinement of exploration targets pose severe challenges because they require the processing of massive amounts of AEM data under high-sampling-rate conditions.

Currently, the interpretation of field-measured AEM data is mainly reliant on imaging technologies and 1D inversion that can ensure the rapid processing of massive amounts of data. Such technologies include the differential resistivity method [4], the modified Sengpiel imaging method [5], the conductivity depth imaging method [6,7,8,9], the tau domain imaging method [10,11], Occam inversion [12,13,14,15,16], the simulated annealing method [17], and Bayesian inversion [18,19]. However, 1D imaging and inversion based on a layered electrical model cannot satisfy the requirements of fine inversion for complex electrical models. Therefore, the 3D AEM inversion of recovered electrical models with fine and complex structures is the key technical means by which AEM data are accurately interpreted. The abovementioned method is also crucial for advancement of near-surface AEM exploration in complex geological conditions. Some studies indicate that the anisotropy of underground media has a serious impact on AEM data [20,21,22,23,24,25,26]. However, current AEM data interpretation is mainly based on isotropic models. When anisotropy is present in underground media, interpreting AEM data based on isotropic models may lead to incorrect interpretations of the measurement data. Therefore, it is necessary to perform AEM inversion based on anisotropy.

Current 3D inversion algorithms are relatively mature and include the nonlinear conjugate gradient method [27,28], the Gauss–Newton method [29,30,31], and the L-BFGS method [32,33,34,35,36]. The nonlinear conjugate gradient method avoids the problems of the Hessian matrix and considerably reduces computational cost, although its convergence speed is slow. The Gauss–Newton method ignores the second-order term of the Hessian matrix and improves the convergence speed. The quasi-Newton method iteratively obtains an approximate Hessian matrix, whereas the L-BFGS method further reduces the need for computational memory and is suitable for large-scale electromagnetic data inversion [37]. Many studies have been conducted on the 3D inversion of AEM data [38,39,40,41,42,43,44,45,46,47,48,49,50,51,52]. Most frequency-domain AEM data acquisition systems use airborne mobile platforms, which simultaneously carry vertical coaxial (VCX) and horizontal coplanar (HCP) coil pairs to acquire data for both orthogonal components. Currently, in AEM data interpretation, only data inversion is generally based on each independent coil pair, which makes it difficult to meet the requirements of fine structural interpretation. Therefore, the development of a method for the joint inversion of the HCP and VCX datasets that can effectively reduce the non-uniqueness of separate 3D inversions and recover more underground structures is necessary.

In this study, we implement the 3D joint inversion of multicomponent frequency-domain AEM data based on the L-BFGS algorithm. First, a 3D forward theory based on unstructured vector finite elements is introduced, followed by the basic theory of L-BFGS inversion. Finally, 5% Gaussian noise is added to the modeling data of the 3D isotropic and anisotropic models to serve as the measured data for separate and joint inversions. The results of the synthetic models show that joint inversion has advantages over separate inversion, i.e., it enables the recovery of finer underground structures.

## 2. Forward Method

The receivers of the AEM system are located very close to the transmitter sources, resulting in the requirement of a fine mesh partition near the transmitter sources in order to meet the accuracy requirements of forward modeling. The receiver setup results in a significant waste of computational resources. Therefore, in this paper, we adopt the method of separating the primary field and the secondary field for forward modeling. The primary field and the secondary field are separated in order to deal with the source term, rendering the fine mesh partition unnecessary [52]. As explained by Zhang et al. [52], by assuming the time harmonic dependence of $e^{i\omega t}$, the governing equation is satisfied by the frequency-domain AEM secondary field:

(1) $$\nabla \times \nabla \times E_{s}+i\omega \mu \sigma E_{s}=-i\omega \mu (\sigma -\sigma_{p})E_{p}$$

where $E_{s}$ and $E_{p}$ represent the background electric field and secondary field, respectively; σ and $\sigma_{p}$ are the model conductivity tensor and background conductivity tensor, respectively; and μ is the magnetic permeability.

(2) $$\sigma =\left[\begin{array}{ccc} \sigma_{x} & & \\ & \sigma_{y} & \\ & & \sigma_{z} \end{array}\right],\;\sigma_{p}=\left[\begin{array}{ccc} \sigma_{x}^{p} & & \\ & \sigma_{y}^{p} & \\ & & \sigma_{z}^{p} \end{array}\right]$$

To ensure the uniqueness of the solution to the electromagnetic field, we need to impose boundary conditions on the electromagnetic diffusion problem. We adopt the Dirichlet first-type boundary condition at the boundary (Γ):

(3) $$E_{s}{|}_{\Gamma }=0$$

Equation (5) can be transformed from Equation (1) (Zhang et al., 2016):

(4) $${∭}_{\Omega }(\nabla \times N^{e}\cdot \nabla \times E_{s})+i\omega \mu \sigma N^{e}\cdot E_{s}d\Omega ={∭}_{\Omega }-i\omega \mu (\sigma -\sigma_{p})N^{e}\cdot E_{p}d\Omega$$

where superscript *e* represents the element number. The calculation region of Equation (4) can be decomposed into a series of tetrahedral elements, and through the integration of Equation (4) within each small element, the coefficient matrix equation within the element can be obtained:

(5) $$K_{}^{e}E_{s}^{e}=S_{}^{e}$$

where $K_{}^{e}$ represents the sum of the density matrix and the stiffness matrix, and $S_{}^{e}$ represents the source vector. By synthesizing the coefficient matrices of all elements into a global matrix, the finite element forward equation of the frequency-domain airborne electromagnetic method can be obtained:

(6) $$KE_{s}^{}=S$$

Equation (6) is solved using the multicore parallel direct solver PARADISO. The secondary magnetic field of the frequency-domain AEM response can then be obtained through Faraday’s law:

(7) $$H_{s}=-\frac{1}{i\omega \mu }\nabla \times E_{s}$$

## 3. Inversion Method

### 3.1. Basic Principles

**Figure 1:**
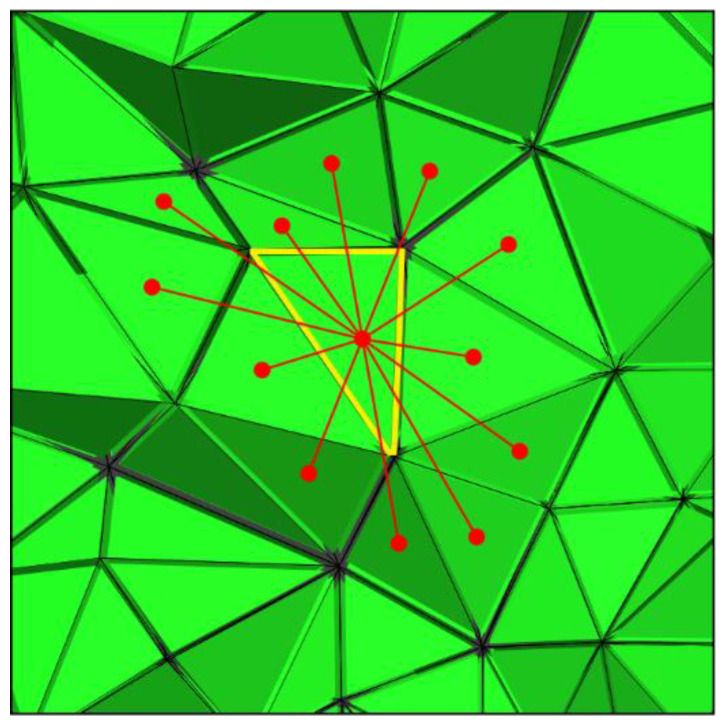
Model constraints for unstructured tetrahedral grids. The red points are the centroids of current (yellow) and adjacent tetrahedrons, and the lines denote the distances between two tetrahedrons.

According to the regularization theory, the geophysical inversion problem can be reduced to a problem of finding the minimum of the following objective function:

(8) $$\Phi (m)=\Phi_{d}(m)+\lambda \Phi_{m}(m)$$

where the data-fitting term ($\Phi_{d}(m)$) is

(9) $$\Phi_{d}(m)=||C_{d}(d-f(m))|{|}^{2}$$

where m represents the model parameter vector, d represents the observed data vector, f(m) represents the forward operator, and $C_{d}$ represents the variance–covariance matrix of the data. The model constraint term ($\Phi_{m}(m)$) is

(10) $$\Phi_{m}(m)=\alpha_{r}\Phi_{r}(m)+\alpha_{s}\Phi_{s}(m)$$

where $\alpha_{r}$ and $\alpha_{s}$ are extra regularization factors, and the expressions for roughness $\Phi_{r}$ and smallness $\Phi_{s}$ are

(11) $$\Phi_{r}=||Rm|{|}^{2}=\sum_{i=1}^{M}V_{i}[\sum_{j=1}^{N(i)}w_{j}[{\left(\frac{\Delta m_{xij}}{d_{ij}}\right)}^{2}+{\left(\frac{\Delta m_{yij}}{d_{ij}}\right)}^{2}+{\left(\frac{\Delta m_{zij}}{d_{ij}}\right)}^{2}]]$$

(12) $$\begin{array}{l} \Phi_{s}=||W_{s}(m-m_{pri})|{|}^{2}\\ =\sum_{i=1}^{M}[\frac{1}{V_{i}}[{\left(m_{xi}-m_{xipri}\right)}^{2}+{\left(m_{yi}-m_{yipri}\right)}^{2}+{\left(m_{zi}-m_{zipri}\right)}^{2}]] \end{array}$$

where $m=[m_{x}^{T},m_{y}^{T},m_{z}^{T}]$, $m_{pri}$ is the prior model, $V_{i}$ represents the volume of the current element, N(i) is the number of neighboring elements for the current element, and $d_{ij}$ represents the distance between the centers of the current element (Figure 1).

(13) $$w_{j}=\frac{V_{j}}{\sum_{k=1}^{N(i)}V_{k}}$$

(14) $$\Delta m_{xij}=m_{xi}-m_{xj},\;\Delta m_{yij}=m_{yi}-m_{yj},\;\Delta m_{zij}=m_{zi}-m_{zj}$$

We set the center point of the element as ($\bar{x_{i}}$,$\bar{y_{i}}$,$\bar{z_{i}}$); then,

(15) $$d_{ij}=\sqrt{{\left(\bar{x_{i}}-\bar{x_{j}}\right)}^{2}+{\left(\bar{y_{i}}-\bar{y_{j}}\right)}^{2}+{\left(\bar{z_{i}}-\bar{z_{j}}\right)}^{2}}$$

Once the objective function is established, the inversion problem is to minimize the objective function. The gradient of the objective function can be expressed as

(16) $$g(m)=\nabla \Phi (m)=\nabla \Phi_{d}(m)+\lambda (\alpha_{r}\nabla \Phi_{r}(m)+\alpha_{s}\nabla \Phi_{s}(m))$$

the gradient of the data-fitting term is

(17) $$\nabla \Phi_{d}(m)=-2J^{T}C_{d}^{T}C_{d}(d-f(m))$$

where ***J*** is the sensitivity matrix: J=∂f(m)/∂m.

Equation (17) can be solved using adjoint forward modeling [53]. The gradients of $\Phi_{r}$ and $\Phi_{s}$ can be directly analytically obtained, and their expressions are as follows:

(18) $$\nabla \Phi_{r}=2\sum_{i=1}^{M}V_{i}[\sum_{j=1}^{N(i)}w_{j}{\left(\frac{1}{d_{ij}}\right)}^{2}(\Delta m_{xij}+\Delta m_{yij}+\Delta m_{zij})]$$

(19) $$\nabla \Phi_{s}=2\sum_{i=1}^{M}[\frac{1}{V_{i}}[(m_{xi}-m_{xipri})+(m_{yi}-m_{yipri})+(m_{zi}-m_{zipri})]]$$

### 3.2. L-BFGS Algorithm

The L-BFGS algorithm does not store all the information of the inverse matrix of the approximate Hessian matrix ($H_{k}$)—it only stores the latest m pairs of $s_{k}$ and $y_{k}$, where m is usually between 3 and 20 (we use 5 in this paper).

(20) $$H_{k+1}=v_{k}^{T}H_{k}v_{k}+\rho_{k}s_{k}s_{k}^{T}$$

Here, $\rho_{k}=1/y_{k}^{T}s_{k}$, $v_{k}=I-\rho_{k}s_{k}y_{k}^{T}$, $s_{k}=m_{k+1}-m_{k}$, $y_{k}=g_{k+1}-g_{k}$, and ***I*** is a unit matrix.

For Equation (20), by keeping the latest m correction pairs in L-BFGS, we can obtain

(21) $$\begin{array}{l} H_{k+1}=(v_{k}^{T}v_{k-1}^{T}\cdots v_{k-m}^{T})H_{0}(v_{k-m}\cdots v_{k-1}v_{k})\\ +(v_{k}^{T}v_{k-1}^{T}\cdots v_{k-m}^{T})(\rho_{0}s_{0}s_{0}^{T})(v_{k-m}\cdots v_{k-1}v_{k})\\ +(v_{k}^{T}v_{k-1}^{T}\cdots v_{k-m}^{T})\rho_{1}s_{1}s_{1}^{T}(v_{k-m}\cdots v_{k-1}v_{k})\\ +\cdots \\ +(v_{k}^{T}v_{k-1}^{T})(\rho_{k-2}s_{k-2}s_{k-2}^{T})(v_{k-1}v_{k})\\ +(v_{k}^{T})\rho_{k-1}s_{k-1}s_{k-1}^{T}(v_{k-1})\\ +\rho_{k}s_{k}s_{k}^{T} \end{array}$$

Due to the complexity of computing $H_{k}$ and the fact that the most important part of model updating is the search direction ($d_{k}$), the double-loop recursive algorithm is used to calculate the search direction ($d_{k}=-H_{k}g_{k}$) [54].

The search step size in the optimization process generally needs to satisfy the following sufficient descent condition:

(22) $$f(m_{k}+\alpha_{k}d_{k})\leq f(m_{k})+c_{1}\alpha_{k}g_{k}^{T}d_{k}$$

and the following curvature condition:

(23) $$g{\left(m_{k}+\alpha_{k}d_{k}\right)}^{T}d_{k}\geq c_{2}g_{k}^{T}d_{k}$$

where $\alpha_{k}$ is the iteration step; $c_{1}$ and $c_{2}$ are constants; $0<c_{1}<c_{2}<1$, and in general, $c_{1}=10^{-4}$ and $c_{2}=0.9$; and k is the iteration number. These two conditions are collectively referred to as Wolfe conditions.

The process of the L-BFGS algorithm is as follows:

(1) We assume the initial model ($m_{1}$) and the initial Hessian matrix ($H_{1}$) (usually the identity matrix). We set the error threshold to ε>0 and the objective root mean square (Rms) to Rms = 0, with iteration number k=1;
(2) We calculate the Rms and gradient of the objective function ($g_{k}$). If Rms<Rms0 or $||g_{k}||<\varepsilon$, the iteration terminates, and the final solution ($m_{k}$) is output; otherwise, continue to the next step;
(3) We use a double-loop recursive algorithm to obtain $d_{k}$;
(4) We search for the iteration step length ($\alpha_{k}$) using the Wolfe conditions and update the model using the following equation: $m_{k+1}=m_{k}+\alpha_{k}d_{k}$;
(5) We set k=k+1 and return to step 2.

## 4. Numerical Experiments

In frequency-domain AEM data processing, data from individual AEM HCP and VCX datasets are commonly processed separately. In this study, we fully exploit the advantages of HCP and VCX datasets in data collection and develop techniques for joint inversion of the two types of data. In the inversion process, the two types of data are sequentially arranged, and the L-BFGS method is used for joint inversion.

### 4.1. Three-Dimensional Frequency-Domain AEM Isotropic Inversion Example

**Figure 2:**
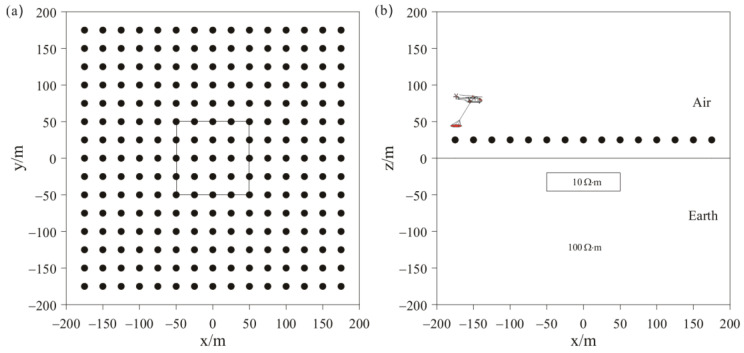
Schematic representations of model 1: (**a**) plan view; (**b**) cross-sectional view.

To test the stability and effectiveness of the inversion algorithm, we added a low-resistivity anomalous body to the background space for the forward modeling, with 5% Gaussian added to the modeling data as the measured data for separate and joint inversions. Figure 2 shows the distribution of anomaly bodies and 225 receivers in model 1, with a survey spacing of 25 m. The resistivity of the background half-space is 100 Ω⋅m, the depth of the anomalous body is 20 m, the size of the anomalous body is 100 m × 100 m × 25 m, and the anomalous resistivity is 10 Ω⋅m. The mesh used in theoretical forward modeling contains 431,223 elements and calculates the HCP and VCX responses for two frequencies: 900 Hz and 5000 Hz. The mesh used in model inversion contains 214,325 elements. The cooling principle is used to change the value of λ. The initial value of λ is 0.01, with additional regularization factors of $\alpha_{r}$ and $\alpha_{s}$ both set to 0.1. The prior model and initial model are set as a half-space with a resistivity of 100 Ω⋅m. To demonstrate the advantage of joint inversion of HCP and VCX data in this study, separate inversions were conducted for HCP and VCX for comparison.

**Figure 3:**
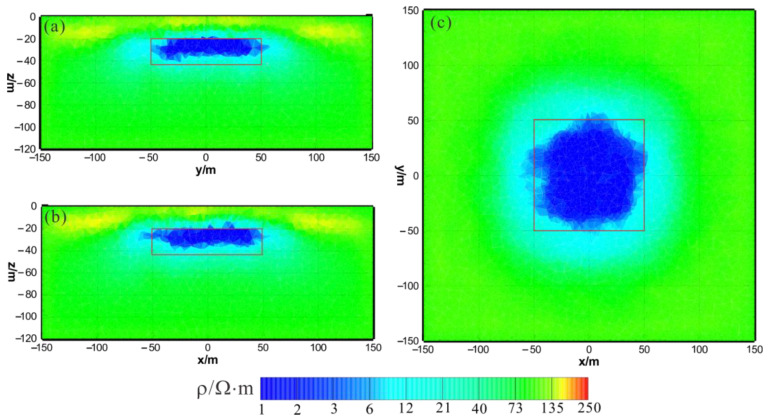
HCP inversion results: (**a**) y–z section (x = 0 m); (**b**) x–z section (y = 0 m); (**c**) x–y section (z = −30 m). The red box area indicates the actual location of the anomalous body.

**Figure 4:**
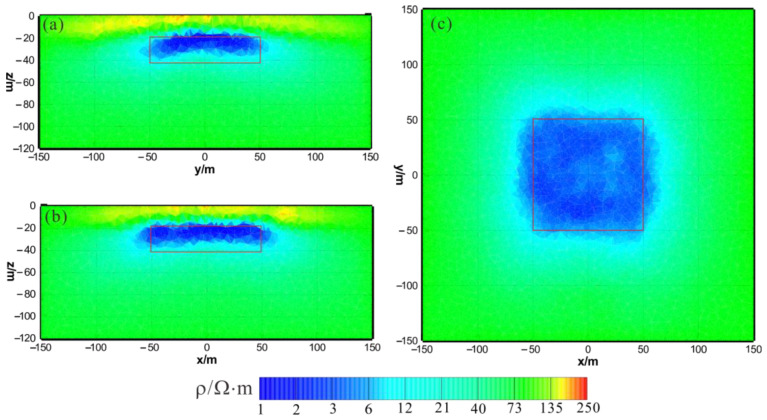
VCX inversion results: (**a**) y–z section (x = 0 m); (**b**) x–z section (y = 0 m); (**c**) x–y section (z = −30 m). The red box area indicates the actual location of the anomalous body.

**Figure 5:**
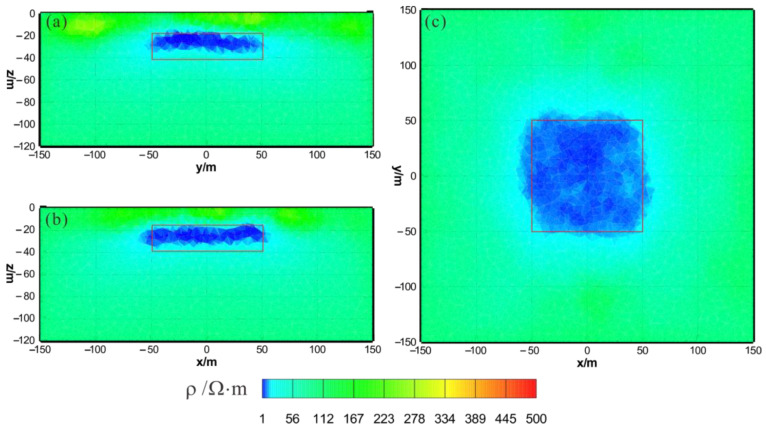
Joint inversion results of HCP and VCX: (**a**) y–z section (x = 0 m); (**b**) x–z section (y = 0 m); (**c**) x–y section (z = −30 m). The red box area indicates the actual location of the anomalous body.

Figure 3 shows the results of the HCP inversion. HCP inversion can roughly recover the true position of the anomalous body. However, the HPC inversion method is subject to two drawbacks: first, the inversion sections show that the estimation of the lower boundary of the anomalous body is not sufficiently accurate; secondly, the shape of the horizontal plane of the anomalous body is deformed and approximates a circular shape smaller than its true extent. The two drawbacks of HCP inversion directly affect the accuracy of inversion interpretation. Figure 4 shows the results of VCX inversion. The lower boundary of VCX inversion is more accurate than HCP inversion, and the contour of the anomalous body is closer to the true value. However, some small, high-resistivity false anomalies can be observed in the VCX inversion slices in the shallow part, which introduces errors to the interpretation of the inversion. Figure 5 shows the joint inversion results of HCP and VCX. Joint inversion is superior to the separate HCP inversion in both lower-boundary and horizontal contours. Moreover, joint inversion does not show false high resistivity anomalies in the shallow part, indicating that it can overcome the limitations of separate HCP and VCX inversions.

**Figure 6:**
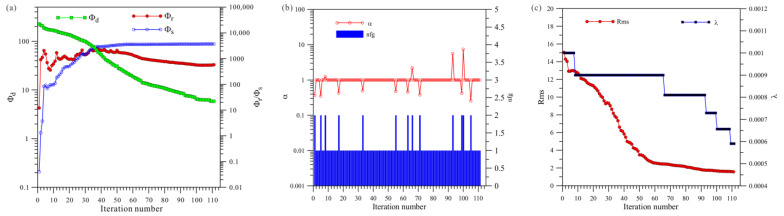
Convergence plots of isotropy HCP inversion: (**a**) fitting term ($\Phi_{d}$), model constraint term ($\Phi_{r}$), and $\Phi_{s}$; (**b**) nfg and search step length (α); (**c**) Rms and regularization factor (λ).

Figure 6a shows a total of 111 iterations of the HCP inversion. The data misfit ($\Phi_{d}$) was reduced from 226.6 to 5.8, and the model’s roughness ($\Phi_{r}$) and $\Phi_{s}$ ultimately stabilized and converged. Figure 6b shows that the HCP inversion changed step sizes 13 times, and Figure 6c shows that the HCP inversion changed the regularization factor six times.

**Figure 7:**
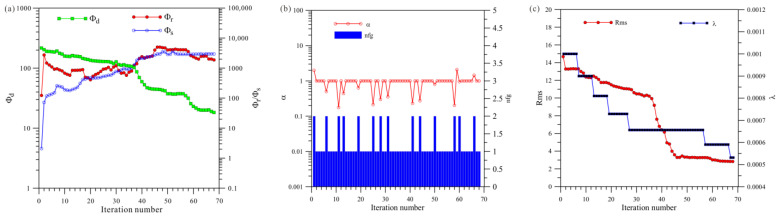
Convergence plots of isotropy VCX inversion: (**a**) fitting term ($\Phi_{d}$), model constraint term ($\Phi_{r}$), and $\Phi_{s}$; (**b**) nfg and search step length (α); (**c**) Rms and regularization factor (λ).

Figure 7a shows a total of 68 iterations of the VCX inversion. The data misfit $\Phi_{d}$ was reduced from 216.4 to 18.2, and the model’s roughness ($\Phi_{r}$) and $\Phi_{s}$ finally stabilized and converged. Figure 7b shows that VCX inversion changed step sizes 14 times, and Figure 7c shows that VCX inversion changed regularization factors seven times.

**Figure 8:**
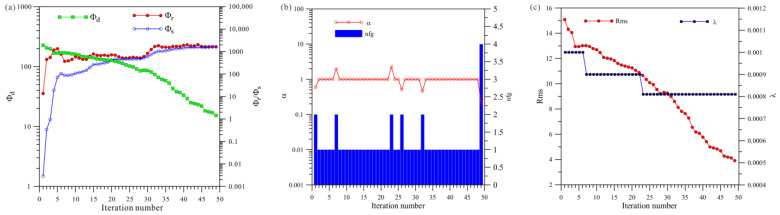
Convergence plots for joint inversion of isotropy HCP and VCX: (**a**) fitting term ($\Phi_{d}$), model constraint term ($\Phi_{r}$), and $\Phi_{s}$; (**b**) nfg and search step length (α); (**c**) Rms and regularization factor (λ).

Figure 8a shows a total of 49 iterations of the joint inversion. The data misfit ($\Phi_{d}$) was reduced from 227.4 to 15.2, and the model’s roughness ($\Phi_{r}$) and $\Phi_{s}$ ultimately stabilized and converged. Figure 8b shows that the joint inversion changed step sizes five times, and Figure 8c shows that the joint inversion changed regularization factors three times.

A comprehensive analysis of Figure 6, Figure 7 and Figure 8 shows that, first, in terms of the number of iterations, the HCP inversion converges slowest, with 111 iterations, whereas the VCX inversion take 68 iterations and the joint inversion is the fastest, only requiring 49 iterations. Secondly, in terms of the number of changes in step size during the inversion process, VCX inversion changes the most (14 times), and HCP inversion changes 13 times, whereas joint inversion only changes five times. The effectiveness of joint inversion is also demonstrated by the smaller number of step size changes. Finally, in terms of the number of changes in the regularization factor, VCX inversion changes the most, followed by HCP inversion, whereas joint inversion changes the least. The fewer the number of changes, the better the stability of the inversion, which also demonstrates the effectiveness of joint inversion in this study.

### 4.2. Three-Dimensional Frequency-Domain AEM Anisotropic Inversion Example

**Figure 9:**
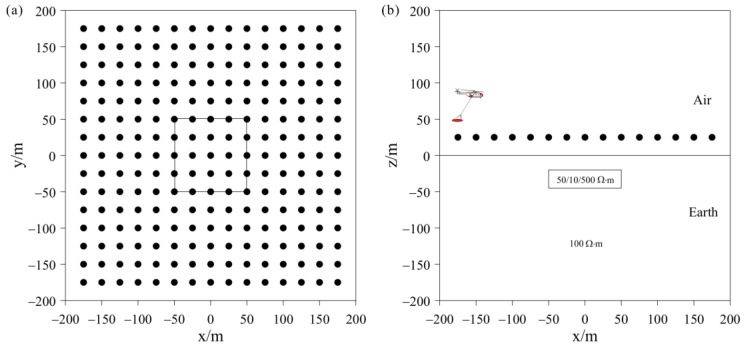
Schematic representations of model 2: (**a**) plan view; (**b**) cross-sectional view.

To test the stability and effectiveness of the triaxial anisotropic inversion algorithm for 3D AEM, in this study, we added an anisotropic anomalous body to the background space for forward modeling, with 5% Gaussian noise added to the modeling data as the measured data for separate and joint inversions. Figure 9 shows the distribution of anomaly bodies and 225 measurement points in model 2, with a survey point spacing of 25 m. The resistivity of the background half-space is 100 Ω⋅m; the depth of the anomalous body is 20 m; the size of the anomalous body is 100 m × 100 m × 25 m; and the principal-axis electrical resistivity values of the anisotropic anomalous body are 50 Ω⋅m for $\rho_{x}$, 10 Ω⋅m for $\rho_{y}$, and 500 Ω⋅m for $\rho_{z}$. The mesh used in theoretical forward modeling contains 445,672 elements and calculates the HCP and VCX responses for two frequencies: 900 Hz and 5000 Hz. The mesh used in model inversion contains 281,242 elements. The cooling principle is used to change the value of λ. The initial value of λ is 0.01, with additional regularization factors of $\alpha_{r}$ and $\alpha_{s}$, both set to 0.1. The prior model and initial model are set as a half-space with a resistivity of 100 Ω⋅m. To demonstrate the advantage of the joint inversion of HCP and VCX data in this study, separate inversions of HCP and VCX were also conducted for comparison.

**Figure 10:**
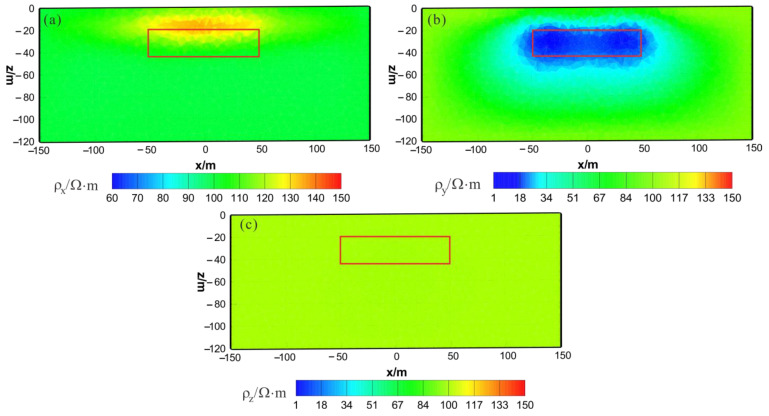
HCP anisotropic inversion results from the x–z section (y = 0 m): (**a**) $\rho_{x}$; (**b**) $\rho_{y}$; (**c**) $\rho_{z}$. The red box area indicates the actual location of the anomalous body.

**Figure 11:**
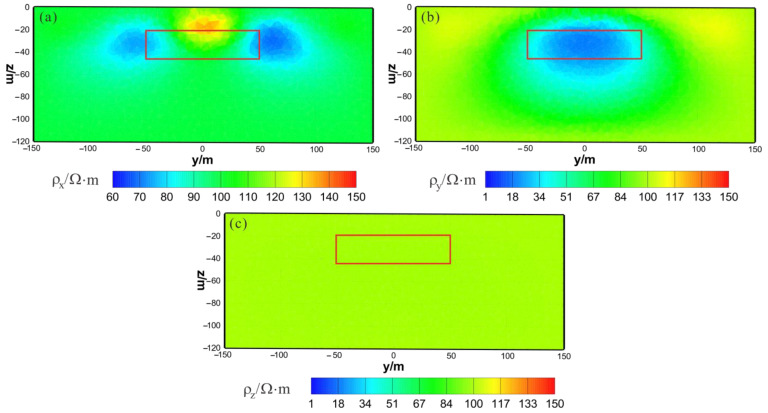
HCP anisotropic inversion results from the y–z section (x = 0 m): (**a**) $\rho_{x}$; (**b**) $\rho_{y}$; (**c**) $\rho_{z}$. The red box area indicates the actual location of the anomalous body.

**Figure 12:**
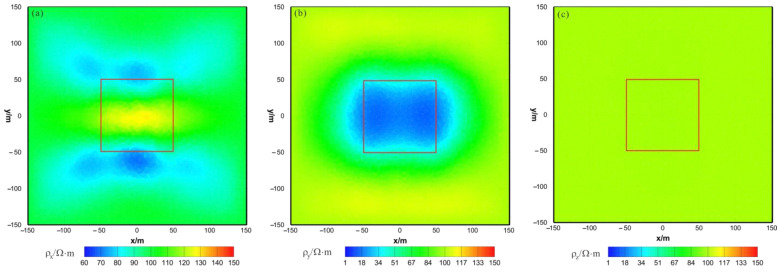
HCP anisotropic inversion results from the x-y section ( z= −30 m): (**a**) $\rho_{x}$; (**b**) $\rho_{y}$; (**c**) $\rho_{z}$. The red box area indicates the actual location of the anomalous body.

As shown in Figure 10a, Figure 11a and Figure 12a, the $\rho_{x}$ of the HCP inversion result has poor recovery. A high-resistivity body appears in the x–z section, with a lower boundary shallower than the actual anomaly body in the z direction. The y–z section shows two low-resistivity bodies and one high-resistivity body, roughly indicating the position of the actual anomaly body. The x–y section shows two low-resistivity bodies and one high-resistivity body, with relatively accurate positioning in the x direction. As shown in Figure 10b, Figure 11b and Figure 12b, the $\rho_{y}$ of the HCP inversion result has a good recovery. The inverted resistivity is close to the true value of 10Ω⋅m. The x–z section accurately depicts the position of the actual anomaly body. The y–z section relatively accurately indicates the positioning in the y direction, but the lower boundary is larger than the actual anomaly body in the z direction, approximately forming an ellipse. The x–y section relatively accurately depicts the positioning in the y direction, but the width is larger than the actual anomaly body in the x direction, approximately forming an ellipse. As show in Figure 10c, Figure 11c and Figure 12c, $\rho_{z}$ is close to the theoretical background resistivity value, and the anomaly is not obvious, indicating that the parameters of anisotropic bodies ($\rho_{z}$) cannot be inverted in 3D anisotropic inversion. Overall, the inversion effect of HCP is acceptable, and the inversion effect of $\rho_{y}$ is satisfactory, roughly restoring the true position of the anomalous body. However, the inversion effect of $\rho_{x}$ is poor, and the recovery of $\rho_{z}$ is difficult.

**Figure 13:**
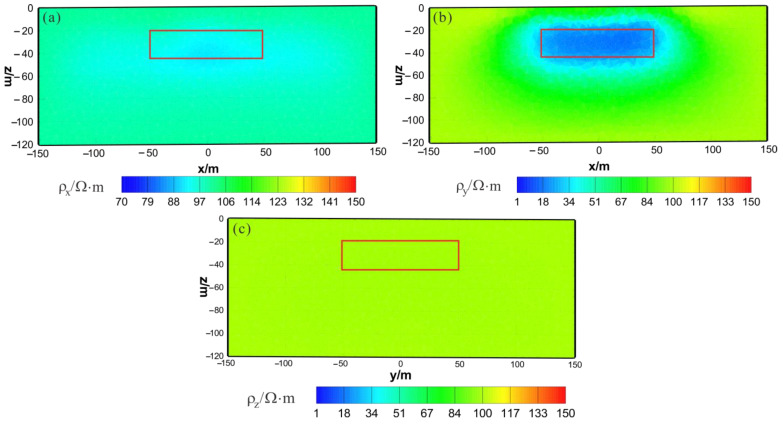
VCX anisotropic inversion results from the x–z section (y = 0 m): (**a**) $\rho_{x}$; (**b**) $\rho_{y}$; (**c**) $\rho_{z}$. The red box area indicates the actual location of the anomalous body.

**Figure 14:**
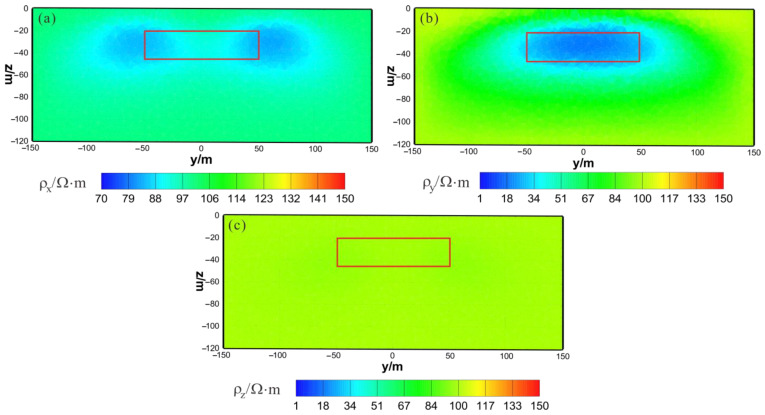
VCX anisotropic inversion results from the y–z section (x = 0 m): (**a**) $\rho_{x}$; (**b**) $\rho_{y}$; (**c**) $\rho_{z}$. The red box area indicates the actual location of the anomalous body.

**Figure 15:**
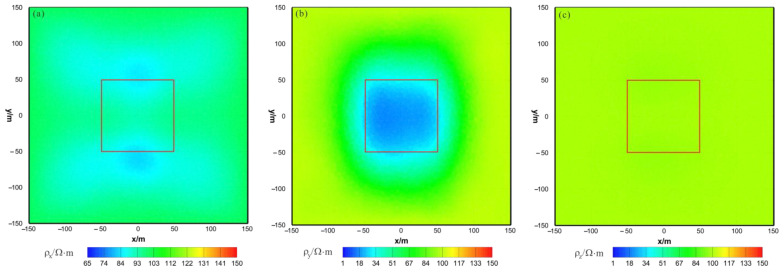
VCX anisotropic inversion results from the x–y section (z = −30 m): (**a**) $\rho_{x}$; (**b**) $\rho_{y}$; (**c**) $\rho_{z}$. The red box area indicates the actual location of the anomalous body.

As shown in Figure 13a, Figure 14a and Figure 15a, the $\rho_{x}$ of the VCX inversion result has moderate recovery. A low-resistivity body appears in the x–z section, with a resistivity of approximately 88 Ω⋅m and a relatively accurate positioning in the x direction and lower boundary larger than the actual model in the z direction. The y–z section shows two low-resistivity bodies with a resistivity of approximately 80 Ω⋅m and relatively accurate positioning in the z direction. The x–y section shows two low-resistivity bodies with a resistivity of approximately 85 Ω⋅m and a relatively accurate positioning in the x direction. As shown in Figure 13b, Figure 14b and Figure 15b, the $\rho_{y}$ of the VCX inversion result has a good recovery. In all three sections, a low-resistivity body appears with a resistivity of approximately 35 Ω⋅m, and the position of the actual abnormal body is well restored. As shown in Figure 13c, Figure 14c and Figure 15c, $\rho_{z}$ is close to the theoretical background resistivity value, and the anomaly is not obvious, indicating that the parameters of anisotropic bodies ($\rho_{z}$) cannot be inverted in 3D anisotropic inversion. Overall, the inversion effect of VCX is acceptable, and the inversion effect of $\rho_{y}$ is satisfactory, roughly restoring the true position of the anomalous body. However, a discrepancy remains between the inverted resistivity and the true resistivity. The inversion effect of $\rho_{x}$ is acceptable, indicating a low-resistivity anomaly, but the recovery of $\rho_{z}$ is difficult.

**Figure 16:**
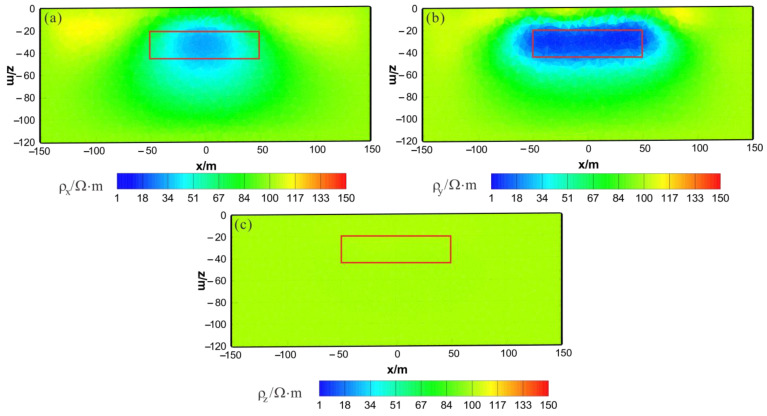
HCP and VCX anisotropic joint inversion results from the x–z section (y = 0 m): (**a**) $\rho_{x}$; (**b**) $\rho_{y}$; (**c**) $\rho_{z}$. The red box area indicates the actual location of the anomalous body.

**Figure 17:**
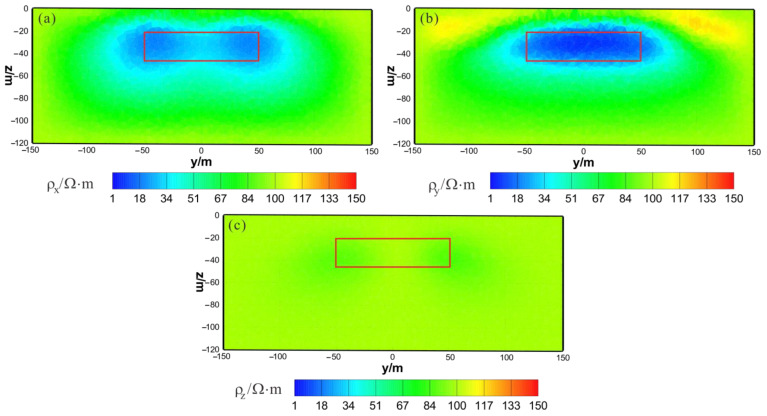
HCP and VCX anisotropic joint inversion results from the y–z section (x = 0 m): (**a**) $\rho_{x}$; (**b**) $\rho_{y}$; (**c**) $\rho_{z}$. The red box area indicates the actual location of the anomalous body.

**Figure 18:**
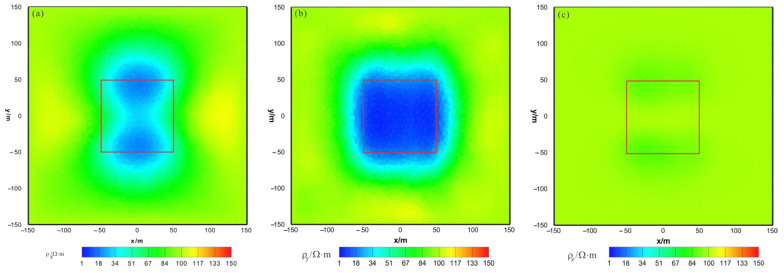
HCP and VCX anisotropic joint inversion results from the x-y section (z = −30 m): (**a**) $\rho_{x}$; (**b**) $\rho_{y}$; (**c**) $\rho_{z}$. The red box area indicates the actual location of the anomalous body.

As shown in Figure 16a, Figure 17a and Figure 18a, the $\rho_{x}$ of the joint inversion result has good recovery. In the x–z section, a low-resistivity body appears with a resistivity of approximately 45 Ω⋅m, with is close to the true value of 50 Ω⋅m. The width is smaller than the actual model in the x direction, and the thickness is greater than the actual model in the z direction, roughly forming a circular shape. The y–z section shows a low-resistivity body with a resistivity of approximately 45  Ω⋅m and a slightly larger width than the actual model in both the x and z directions. The x–y section shows a low-resistivity body with a resistivity of approximately 45 Ω⋅m, a relatively accurate positioning in the x direction, and a wider width in the y direction than the actual model. As shown in Figure 16b, Figure 17b and Figure 18b, the $\rho_{y}$ of the joint inversion result has good recovery. The inverted resistivity is very close to the true value of 10 Ω⋅m, and position of the actual abnormal body is restored well. As shown in Figure 16c, Figure 17c and Figure 18c, $\rho_{z}$ is close to the theoretical background resistivity value, and the anomaly is not obvious, indicating that the parameters of anisotropic bodies ($\rho_{z}$) cannot be inverted in 3D anisotropic inversion. Overall, the joint inversion effect is very good, with a positive inversion effect of $\rho_{y}$, which can recover the true position of the anomalous body. The inverted resistivity is close to the true resistivity. The inversion effect of $\rho_{x}$ is also acceptable, roughly restoring the true position of the anomalous body, with little difference in the inverted resistivity and the true resistivity. However, the recovery of $\rho_{z}$ is difficult.

Under the condition of triaxial anisotropy, $\rho_{z}$ has difficulty recovering and obtaining a consensus conclusion in time-domain 3D AEM anisotropy inversion [51]. As the electromagnetic method is more sensitive for low resistivity, the inversion results of $\rho_{y}$ with the lowest resistance are the best, followed by the inversion results of $\rho_{x}$ with the lowest resistance.

**Figure 19:**
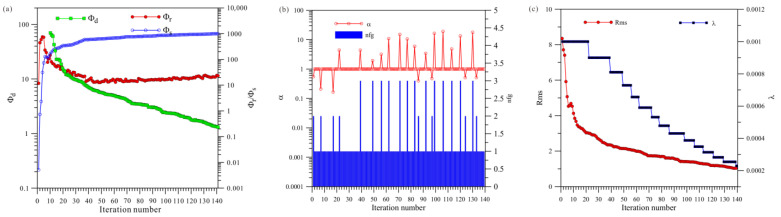
Convergence plots of anisotropy HCP inversion: (**a**) fitting term ($\Phi_{d}$), model constraint term ($\Phi_{r}$), and $\Phi_{s}$; (**b**) nfg and search step length (α); (**c**) Rms and regularization factor (λ).

Figure 19a shows a total of 141 iterations of HCP inversion. The data misfit ($\Phi_{d}$) was reduced from 69.8 to 1.31, and the model’s roughness ($\Phi_{r}$) and $\Phi_{s}$ ultimately stabilized and converged. Figure 19b shows that HCP inversion changed step sizes 21 times, and Figure 19c shows that HCP inversion changed regularization factors 15 times.

**Figure 20:**
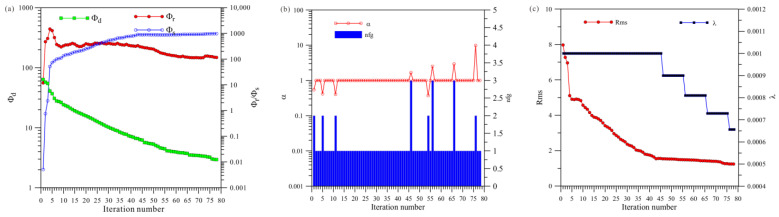
Convergence plots of anisotropy VCX inversion: (**a**) fitting term ($\Phi_{d}$), model constraint term ($\Phi_{r}$), and $\Phi_{s}$; (**b**) nfg and search step length (α); (**c**) Rms and regularization factor (λ).

Figure 20a shows a total of 78 iterations of VCX inversion. The data misfit $\Phi_{d}$ was reduced from 63.6 to 2.9, and the model’s roughness ($\Phi_{r}$) and $\Phi_{s}$ finally stabilized and converged. Figure 20b shows that HCP inversion changed step sizes eight times, and Figure 20c shows that HCP inversion changed regularization factors five times.

**Figure 21:**
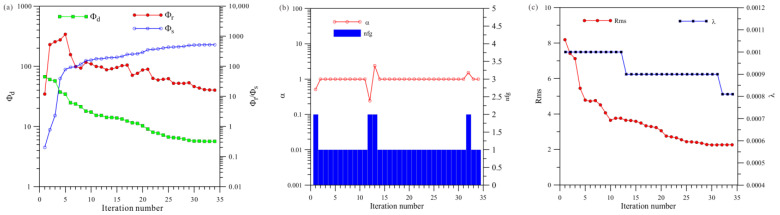
Convergence plots for joint inversion of anisotropy HCP and VCX: (**a**) fitting term ($\Phi_{d}$), model constraint term ($\Phi_{r}$), and $\Phi_{s}$; (**b**) nfg and search step length (α); (**c**) Rms and regularization factor (λ).

Figure 21a shows a total of 34 iterations of the joint inversion. The data misfit ($\Phi_{d}$) was reduced from 67.1 to 5.6, and the model’s roughness ($\Phi_{r}$) and $\Phi_{s}$ ultimately stabilized and converged. Figure 21b shows that joint inversion changed step sizes four times, and Figure 21c shows that joint inversion changed regularization factors three times.

A comprehensive analysis of Figure 19, Figure 20 and Figure 21 shows that, first, in terms of the number of iterations, the HCP inversion converges slowest, with 141 iterations; the VCX inversion takes 78 iterations; and the joint inversion example is the fastest, only requiring 34 iterations. Secondly, in terms of the number of changes in step size during the inversion process, HCP inversion changes the most (21 times), VCX inversion changes eight times, and joint inversion only changes four times. The effectiveness of joint inversion is also demonstrated by the smaller number of step size changes. Finally, in terms of the number of changes in the regularization factor, HCP inversion changes the most, followed by VCX inversion, whereas joint inversion changes the least. The fewer the number of changes, the better and more stable the inversion, which also demonstrates the effectiveness of joint inversion in this study.

## 5. Conclusions

A 3D AEM multicomponent joint inversion algorithm for both isotropic and anisotropic cases based on the finite element and L-BFGS methods was developed in this study. Synthetic models prove the effectiveness of joint inversion compared to the separate inversions in both simply isotropic and anisotropic cases. This study revealed that for isotropic model cases, separate HCP and VCX inversions can effectively recover the distribution and magnitude of resistivity in the model, with some shortcomings. Joint inversion can effectively alleviate the shortcomings of separate inversion, achieving improved inversion results. Because the electromagnetic method is more sensitive for low resistivity, for anisotropic model cases, separate HCP and VCX inversions can effectively recover the distribution and magnitude of $\rho_{y}$ in the model, although the inversion results for $\rho_{x}$ are not as satisfactory. Joint inversion can effectively recover the distribution and magnitude of both $\rho_{x}$ and $\rho_{y}$ in the model, resulting in improved inversion results. Based on the inversion results for isotropic and anisotropic models, joint inversion achieves better results than separate inversion, and can provide a new approach for fine structural interpretation of frequency-domain AEM data.

## Data Availability

Data associated with this research are available and can be obtained by contacting the corresponding author.
